# Reduced Skeletal Muscle Volume and Increased Skeletal Muscle Fat Deposition Characterize Diabetes in Individuals after Pancreatitis: A Magnetic Resonance Imaging Study

**DOI:** 10.3390/diseases8030025

**Published:** 2020-07-01

**Authors:** Andre E. Modesto, Juyeon Ko, Charlotte E. Stuart, Sakina H. Bharmal, Jaelim Cho, Maxim S. Petrov

**Affiliations:** School of Medicine, University of Auckland, Auckland 1023, New Zealand; amod428@aucklanduni.ac.nz (A.E.M.); ju.ko@auckland.ac.nz (J.K.); cstu166@aucklanduni.ac.nz (C.E.S.); s.bharmal@auckland.ac.nz (S.H.B.); j.cho@auckland.ac.nz (J.C.)

**Keywords:** diabetes, pancreatitis, skeletal muscle, ectopic fat, insulin traits, magnetic resonance imaging

## Abstract

Background: Skeletal muscle has been implicated in the pathogenesis of type 2 diabetes but it has never been investigated in diabetes after pancreatitis. The aim was to investigate the relationship between psoas muscle volume (PMV) and diabetes in individuals after pancreatitis, as well as its associations with ectopic fat phenotypes and insulin traits. Methods: Individuals after an attack of pancreatitis and healthy individuals were studied in a cross-sectional fashion. All participants underwent magnetic resonance imaging, based on which PMV, skeletal muscle fat deposition (SMFD), as well as liver and intra-pancreatic fat depositions were derived. Fasting and postprandial blood samples were collected to calculate indices of insulin sensitivity and secretion. Linear regression analyses were conducted, adjusting for possible confounders (age, sex, body composition, comorbidities, use of insulin, and others). Results: A total of 153 participants were studied. PMV was significantly decreased in the diabetes group compared with healthy controls (β = −30.0, *p* = 0.034 in the most adjusted model). SMFD was significantly inversely associated with PMV (β = −3.1, *p* < 0.001 in the most adjusted model). The Matsuda index of insulin sensitivity was significantly directly associated with PMV (β = 1.6, *p* = 0.010 in the most adjusted model). Conclusions: Diabetes in individuals after pancreatitis is characterized by reduced PMV. Reduced PMV is associated with increased SMFD and decreased insulin sensitivity in individuals after pancreatitis.

## 1. Introduction

Diabetes is a common multifaceted disease, with at least 450 million people affected by it worldwide [1]. Further, there are more than 370 million people with impaired glucose tolerance. The global healthcare cost of managing people with diabetes is estimated to be at least US$ 850 billion [1]. These data cover all types of the heterogeneous disease [2]. Post-pancreatitis diabetes mellitus (PPDM) is the second most common type of new-onset diabetes in adults [3]. PPDM individuals are more likely to have poor glycemic control compared with those with type 2 diabetes mellitus (T2DM) [4]. Individuals with PPDM also have a significantly higher risk of mortality and hospitalization compared with T2DM individuals [5]. Further, the effectiveness of common glucose-lowering medications is different in PPDM compared with T2DM [6,7].

Decreased insulin sensitivity is an established characteristic of diabetes in individuals after pancreatitis [8,9,10]. They are also characterized by increased visceral fat volume and intra-pancreatic fat deposition, which contribute to changes in insulin sensitivity [11,12]. Another possible contributor is skeletal muscle as it accounts for up to 80% of glucose disposal in the postprandial state and skeletal muscle fatty acid oxidation covers about 90% of the energy requirements in the rested state [13]. The association between skeletal muscle size, including psoas muscle volume (PMV)—a validated surrogate for overall skeletal muscle size [14], and indices of insulin sensitivity has been demonstrated in T2DM [15]. However, it remains unknown whether skeletal muscle size is associated with diabetes in individuals after pancreatitis. Furthermore, although sarcopenic obesity has been recognized as a condition that is strongly associated with an increased risk of T2DM [16] and skeletal muscle fat deposition (SMFD) has been associated with T2DM [17], whether this holds true in diabetes after pancreatitis has never been studied.

The primary aim of this study was to investigate the relationship between diabetes status and PMV in individuals after pancreatitis. The secondary aim was to investigate the relationship between PMV and insulin traits. The tertiary aim was to investigate the relationship between PMV and ectopic fat depots.

## 2. Materials and Methods

### 2.1. Study Design

This was a cross-sectional study nested into prospective longitudinal study as part of the ARIES (Analytic moRphomics In pancrEatic diSeases) project. Adults (18 years of age or older) prospectively diagnosed with pancreatitis were recruited. The diagnosis of acute pancreatitis was established based on the presence of at least two of the following three criteria:

1. Laboratory: Serum amylase and/or lipase at least three times the upper reference limits;
2. Clinical: Pain suggestive of acute pancreatitis (AP); and
3. Radiologic: Characteristic imaging findings of AP.

The diagnosis of chronic pancreatitis was established based on the presence of parenchymal or ductal calcifications on magnetic resonance (MR) imaging or computed tomography and/or Cambridge grade greater than or equal to 3. The exclusion criteria were detailed elsewhere [18,19]. The groupings were based on the American Diabetes Association guidelines, with normoglycemia defined as fasting plasma glucose < 5.6 mmol/L and glycated hemoglobin (HbA1c) < 39 mmol/mol; prediabetes—fasting plasma glucose between 5.6 mmol/L and 6.9 mmol/L and/or HbA1c between 39 and 47 mmol/mol; and diabetes—fasting plasma glucose > 7.0 mmol/L and/or HbA1c > 4.8 mmol/mol [20].

Healthy participants were also enrolled as the control group. The eligible participants had no personal and family history of diseases of the exocrine pancreas or diabetes; no personal history of malignancy, coeliac disease, cystic fibrosis; no upper abdominal pain and nausea at the time of recruitment; no history of inflammatory diseases or acute infections requiring medical care or evaluation in the six months before the study; and body mass index (BMI) above 18.5 kg/m^2^. Informed consent was provided by all participants. The study protocol conformed to the ethical guidelines of the 1975 Declaration of Helsinki.

### 2.2. MR-Derived Variables 

**Figure 1:**
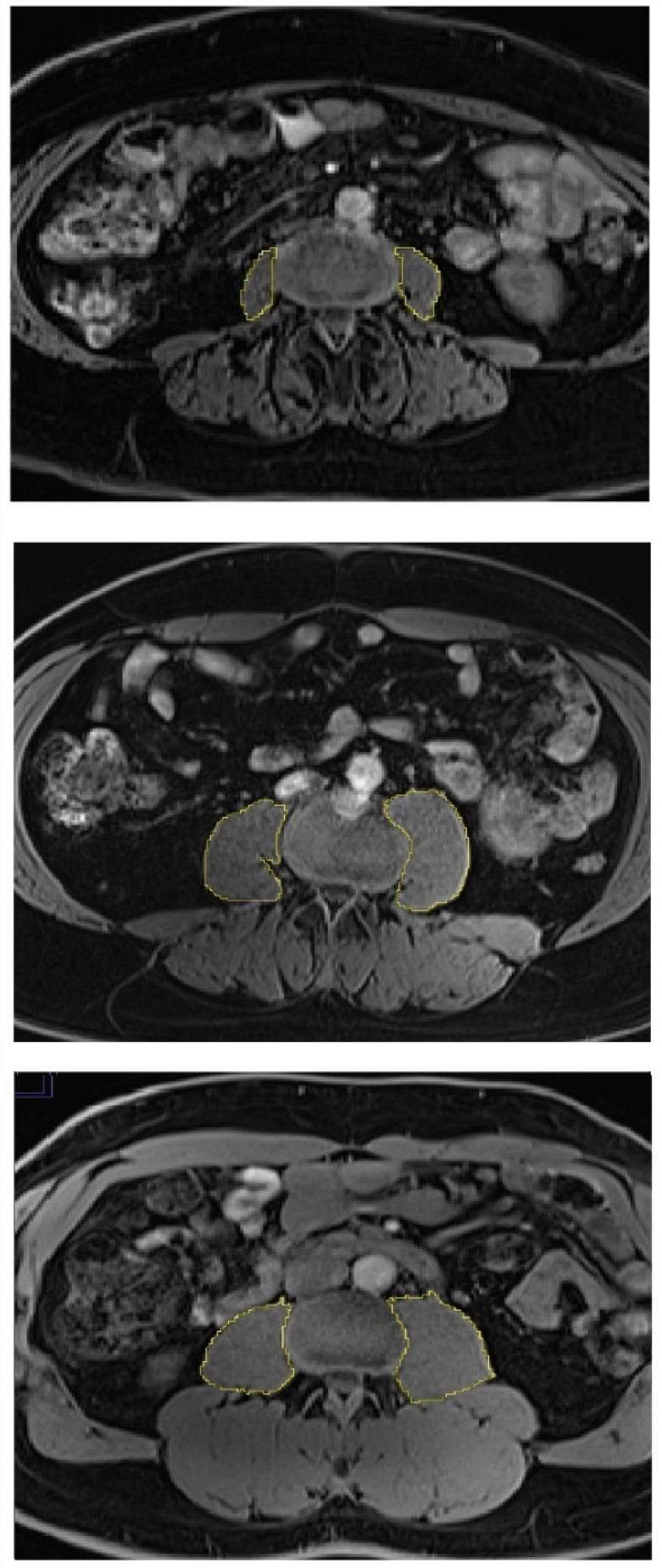
Skeletal muscle size in individuals with diabetes (upper panel), prediabetes (middle panel), and normoglycemia (lower panel). Psoas muscle was used for skeletal muscle size measurements.

**Figure 2:**
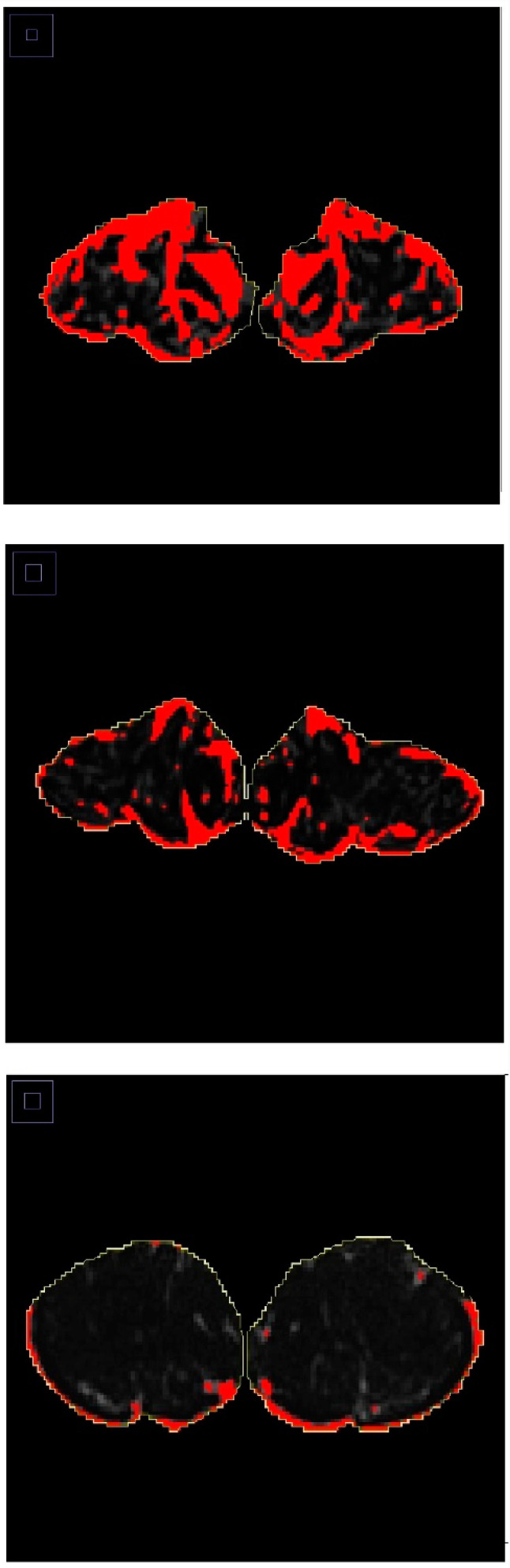
Skeletal muscle fat deposition in individuals with diabetes (upper panel), prediabetes (middle panel), and normoglycemia (lower panel). Paraspinous muscles were used for skeletal muscle fat deposition measurements.

Abdominal MR scans were performed in all participants using 3.0 Tesla MAGNETOM Skyra^®^ MR scanner (Siemens, Erlangen, Germany). Details of the protocol were described elsewhere [21]. PMV was calculated in cm3 by measuring the cross-sectional area of the left and right psoas major muscle from the second lumbar vertebral level to the fifth lumbar vertebral level and multiplying it by the thickness of MR slices (0.3 cm), as described in detail elsewhere [22]. Representative measurements of PMV are displayed in Figure 1. SMFD was determined from the cross-sectional area (in pixels) of the left and right paraspinous muscles (i.e., iliocostalis, longissimus, and multifidus) at the lower endplate of the third lumbar vertebra (total paraspinous area), as described in detail elsewhere [23]. Representative measurements of SMFD are displayed in Figure 2.

In addition, four extra-muscular fat phenotypes were quantified. Segmentation of subcutaneous and visceral fat compartments was performed from the second to the fifth lumbar level, as described in detail elsewhere [24]. Visceral to subcutaneous (V/S) fat volume ratio was then calculated by dividing the visceral fat volume by the subcutaneous fat volume. The above volumes were measured in liters. Intra-pancreatic fat deposition was measured using out-of-phase images, as described in detail elsewhere [18]. Liver fat deposition was measured using single-voxel spectroscopy, as described in detail elsewhere [25]. Two raters, blinded to group allocation and participant characteristics, did the measurements.

### 2.3. Laboratory Variables 

Fasting venous blood samples were analyzed for HbA1c, fasting plasma glucose, and pancreatic amylase at the time of MR acquisition. HbA1c (mmol/mol) was measured using a boronate affinity chromatography assay (Trinity Biotech, Wicklow, Ireland). Fasting serum glucose was measured using an enzymatic colorimetric assay (F. Hoffmann-La Roche). Pancreatic amylase was measured as a proxy for exocrine pancreatic function using the Reflotron^®^ Plus reflectance photometer (Roche ^®^, Basel, Switzerland). Insulin was measured using the MILLIPLEX MAP Human metabolic hormone magnetic bead panel. The indices of insulin secretion included homeostasis model assessment (HOMA)-β in fasting state, and Stumvoll index, insulinogenic index 30′, and insulinogenic index 60′ in postprandial state (after a standardized mixed meal test, as described elsewhere [26]). The indices of insulin sensitivity included HOMA-insulin sensitivity (IS), Raynaud index, 1/fasting insulin in fasting state, and Matsuda index in postprandial state.

### 2.4. Other Variables

A previously validated standardized questionnaire was administered at the time of MR acquisition. The collected data were alcohol consumption (average weekly alcohol consumption in standard alcohol units), smoking status (participants were grouped into never, former, light (<20 cigarettes/day), moderate (20–39 cigarettes/day), and heavy smokers (≥40/cigarettes/day)), and physical activity status (less or more than 2.5 h of physical activity per week) [27,28]. Other variables included the age-adjusted Charlson comorbidity index (ACCI), with a score above 4 being indicative of high risk for pancreatitis comorbidity [29,30]. The use of insulin as a glucose-lowering medication was verified at the time of MR image acquisition.

### 2.5. Statistical Analyses

Significance of the differences in baseline characteristics between the study groups was investigated using analysis of variance (continuous variables) and chi-squared tests (categorical variables). To investigate the differences in PMV between each of the post-pancreatitis group (normoglycemia, prediabetes, and diabetes) and the healthy controls group (i.e., the primary aim), five linear regression models were constructed, with healthy controls as the reference group. Model 1 was unadjusted. Model 2 was adjusted for age and sex. Model 3 was adjusted for the variables included in model 2 as well as V/S fat ratio and physical activity status. Model 4 was adjusted for the variables included in model 3 as well as smoking status and alcohol consumption. Model 5 was adjusted for the variables included in model 4 as well as ACCI, pancreatic amylase, and the use of insulin.

To investigate the associations between PMV and insulin traits (indices of insulin secretion and sensitivity) in the overall cohort (i.e., the secondary aim), linear regression analysis was conducted. To investigate the associations between PMV and ectopic fat deposition phenotypes (liver, intra-pancreatic in the overall cohort (i.e., the tertiary aim)), linear regression analysis was conducted. In these analyses, the models described above were used. Data from the linear regression models were presented as beta coefficients, standard errors, *R²* metrics of the overall model, and *p*-values. Statistical significance was set as two-sided *p* < 0.05 in all analyses. All analyses were performed using SPSS for Windows version 25 (SPSS Inc.).

## 3. Results

### 3.1. Study Characteristics

A total of 114 individuals after pancreatitis were included in the study, comprising 76 men and 38 women. The median (interquartile range (IQR)) age was 55 (44–66) years, the median (IQR) BMI was 27.2 (22.8–31.6) kg/m², and the median (IQR) HbA1c was 38 (35–41) mmol/mol. Thirty-three participants had normoglycemia, 55 had prediabetes, and 26 had diabetes. In individuals after pancreatitis, MR images were acquired at 25.7 (6.0–45.4) months since the last attack. Thirty-nine healthy controls, comprising 20 men and 19 women, were also included. Their median (IQR) age was 49 (39–59) years, the median (IQR) BMI was 24.1 (20.4–27.8), and the median (IQR) HbA1c was 33 (31–35) mmol/mol. Other characteristics are presented in Table 1.

### 3.2. Psoas Muscle Volume in the Study Groups

In the overall cohort, the mean (standard deviation (SD)) PMV was 254 ± 70 cm^³^. The difference in PMV between each of the post-pancreatitis groups and the healthy controls group was not statistically significant in the unadjusted model (*p* = 0.149, 0.834, and 0.447, for the normoglycemia, prediabetes, and diabetes groups, respectively). The difference in PMV was statistically significant in the diabetes group in all the adjusted models (*p* = 0.001, 0.023, 0.025, and 0.0.34, respectively). The difference in PMV was not statistically significant in the prediabetes groups in all the adjusted models (Table 2).

### 3.3. Associations between Psoas Muscle Volume and Insulin Traits

In the overall cohort, mean (SD) values of the indices of insulin secretion were as follows: HOMA-β—150.3 ± 78.1, Stumvoll index—3512 ± 11,863, insulinogenic index 30′—0.52 ± 1.11, and insulinogenic index 60′—0.64 ± 1.14. None of the indices of insulin secretion was significantly associated with PMV (Table 3).

Mean (SD) values of the indices of insulin sensitivity were as follows: HOMA-IS—0.81 ± 0.48, Reynaud index—0.65 ± 1.04, 1/Fasting insulin—0.11 ± 0.18, and Matsuda index—9.30 ± 8.73. The Matsuda index was significantly associated with PMV in all the adjusted models (*p* = 0.005, 0.016, 0.022, and 0.010 in models 2 through 5, respectively). The other indices of insulin sensitivity were not significantly associated with PMV (Table 3).

### 3.4. Associations between Psoas Muscle Volume and Ectopic Fat

**Figure 3:**
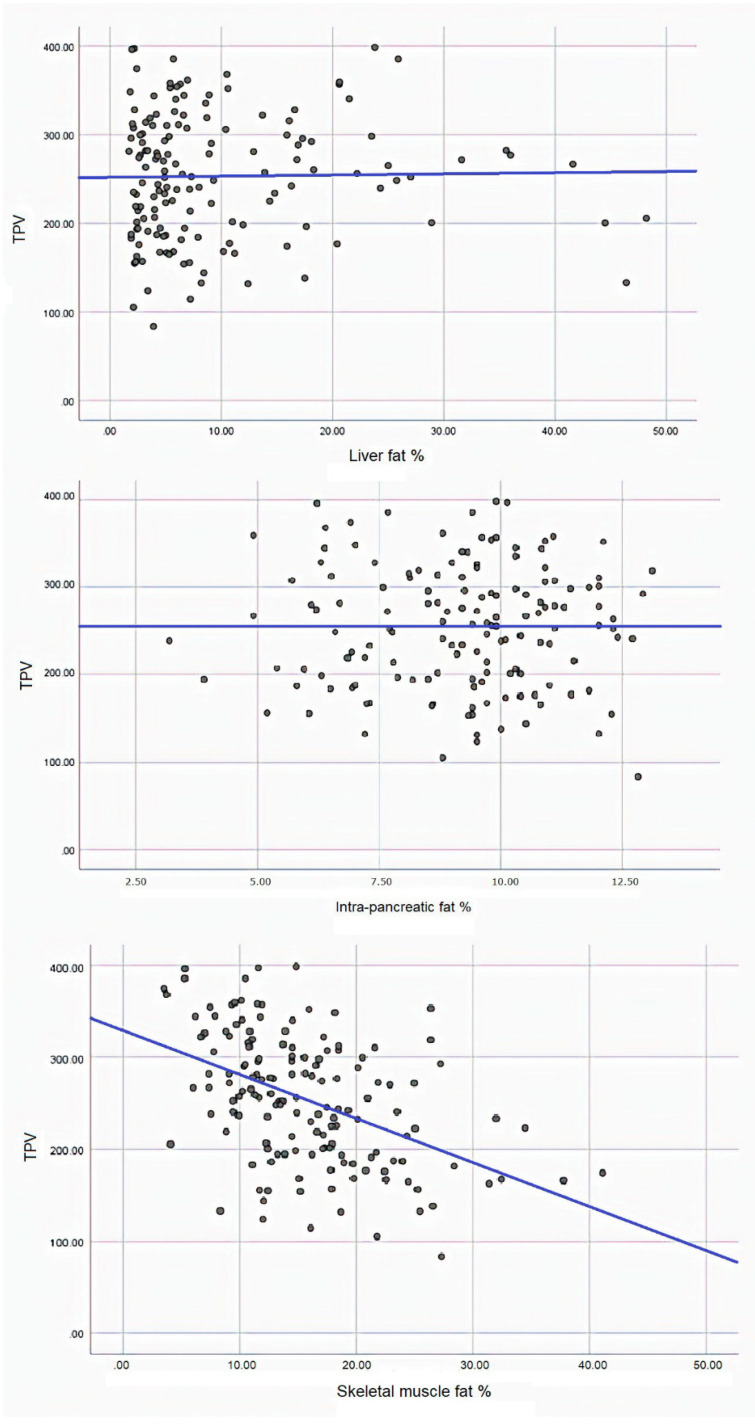
Relationship between psoas muscle volume and liver fat deposition (upper panel), intra-pancreatic fat deposition (middle panel), and skeletal muscle fat deposition (lower panel).

In the overall cohort, mean (SD) SMFD was 15.75% ± 6.77%, intra-pancreatic fat deposition—9.17% ± 2.00%, and liver fat deposition—9.67% ± 9.48%. The associations between PMV and ectopic fat phenotypes are presented in Figure 3. While SMFD contributed 21.5% to variance in PMV, liver and intra-pancreatic fat deposition did not contribute to it (R^2^ = 0% for each). SMFD was inversely associated with PMV in all the models (*p* < 0.001, <0.001, 0.003, 0.002, and <0.001 in models 1 through 5, respectively). Liver fat deposition and intra-pancreatic fat deposition were not significantly associated with PMV in any of the studied models (Table 4).

## 4. Discussion

To date, the investigations of skeletal muscle size in people with diabetes have yielded conflicting results. While some authors reported an increase in skeletal muscle size in diabetes, others found a decrease in it [31,32,33,34]. One reason for this discrepancy is the use of various imaging modalities. Studies that used dual-energy X-ray absorptiometry (DXA) to estimate lean mass generally report an increase in skeletal muscle mass in diabetes, whereas studies that used MR imaging generally report a decrease in skeletal muscle mass in diabetes. For example, a DXA study compared body composition between people with normal glucose tolerance, impaired glucose tolerance, and T2DM patients and found a 2.5% increase in skeletal muscle mass in T2DM individuals as compared with normoglycemia individuals (although a notable finding was a significant decrease in the total lean mass to fat mass ratio) [31]. Another DXA study demonstrated that individuals with T2DM had a 4–12% greater lean muscle mass compared with individuals without diabetes [32], despite worse or comparable muscle strength (in men and women, respectively). However, that study excluded participants with comorbidities as well as individuals who were not fit for strength and endurance tests. By contrast, an MR study found a 19% decrease in proximal leg muscle volume in T2DM individuals (compared with matched healthy controls) [33]. Another MR study found a 7% decrease in muscle mass among T2DM participants with neuropathy (compared with T2DM participants without neuropathy) [34]. Similarly, the present study found a 10% decrease in muscle volume in the diabetes group (compared with healthy controls) in the most adjusted model. MR, alongside computed tomography, is considered the gold standard in measuring skeletal muscle size [35]. In comparison with computed tomography, MR has the advantage of avoiding exposure to radiation and, therefore, it can be used in large body segments, enabling the measurements of volume instead of cross-sectional area. A study found volume measurements to be 10% more accurate than area measurements [36]. To the best of our knowledge, the present study was the first to investigate MR-derived volume (not area) of a muscle of the trunk (not muscles of the extremities) in individuals with diabetes. Further, for the first time, PMV measurements in the whole study cohort were done independently by two raters blinded to participants’ characteristics and study group allocation and the inter-rater reliability was excellent (data not shown).

Given that skeletal muscle is considered the primary site for insulin-stimulated glucose disposal, investigating the relationship between skeletal muscle size and insulin traits is important. This was the first study to investigate the association between skeletal muscle size and insulin traits in participants with abnormal glucose metabolism in a comprehensive manner (covering a range of indices of both insulin secretion and insulin sensitivity, determined in both fasting and postprandial states). An earlier study in individuals with gestational diabetes investigated the relationship between a fasting insulin secretion index (HOMA-β) and appendicular skeletal muscle mass/total fat mass ratio and found no significant association [37]. Similarly, the present study found no significant association between PMV and HOMA-β. To the best of our knowledge, this was the first study to investigate the relationship between skeletal muscle volume and postprandial indices of insulin secretion (i.e., Stumvoll index, insulinogenic index 30ʹ, and insulinogenic index 60ʹ) in individuals with diabetes and no significant association was found. Another earlier cross-sectional study reported an inverse association between skeletal muscle index and a fasting insulin sensitivity index (HOMA-IR) [38]. A 10-year longitudinal study also found an inverse relationship between HOMA-IR and thigh muscle area [39]. The present study used HOMA-IS and found no significant association with PMV. One reason behind the conflicting findings might be the imaging modalities used (MR imaging in the present study as opposed to bioimpedance and computed tomography in the above-mentioned studies). The other reason might relate to the body region studied (PMV in the present study as opposed to whole-body and thigh cross-sectional area in the above-mentioned studies). While none of the fasting indices of insulin sensitivity was significantly associated with skeletal muscle size in the present study, we did find a significant association between a postprandial index of insulin sensitivity (Matsuda index) and PMV in all adjusted models (*p* = 0.05, 0.016, 0.022, and 0.010 in models 2 through 5, respectively). Similarly, the study in individuals with gestational diabetes mentioned above found a significant association between the Matsuda index and appendicular skeletal muscle mass/total fat ratio [37].

To better understand the impairment of peripheral insulin sensitivity in individuals with diabetes, we investigated three ectopic fat depots (and adjusted for V/S fat volume ratio). The present study found an inverse association between SMFD (but not liver or intra-pancreatic fat deposition) and PMV in all models. Skeletal muscle fat contributed 21.5% to variance in PMV (overall cohort) in the unadjusted model. A mean SMFD of 11.1% and 15.1% was observed in the normoglycemia and diabetes group, respectively. Similarly, an earlier MR study of 349 participants investigated abdominal muscle fat deposition in healthy controls versus T2DM and found it to be 10.1% and 13.1%, respectively [40]. The exact mechanisms behind increased SMFD in individuals with diabetes are not completely understood. However, it is generally believed that the impairment of glucose metabolism in skeletal muscles and the resulting impaired insulin sensitivity occur through binding of the insulin molecule to the insulin receptor. This happens in a cascade of events resulting in the translocation of the insulin-sensitive glucose transporter protein 4 (GLUT4) to the plasma membrane, which leads to facilitated diffusion of glucose into the cell [41]. Mitochondrial dysfunction also contributes to insulin resistance through activation of protein kinase C, point alterations in mitochondrial DNA, as well as oxidative stress and excessive free radical generation [42]. Last, the crosstalk between myokines (e.g., interleukin-6, irisin, and fibroblast growth factor 21) and adipokines (e.g., adiponectin, adipocyte fatty acid-binding protein) is thought to contribute to SMFD in diabetes [43].

The present study should be considered in light of the following limitations. First, there is a possibility of unmeasured confounders. Specifically, malnutrition due to insufficient caloric intake may affect both muscle size and diabetes status. Although data on caloric intake were not analyzed in the present study, participants with BMI <18.5 kg/m^2^ were excluded from the study a priori. Another confounder that might affect both muscle size and diabetes status is the long-term use of corticosteroids [44], which was an exclusion criterion in the present study. Moreover, serum levels of cortisol did not play a role in metabolic changes after pancreatitis in our earlier study [45]. The same study also demonstrated no contribution of growth hormone, which stimulates insulin-like growth factor 1 (usually presented in low levels in individuals with reduced muscle size). Second, kidney disease may affect muscle size. In particular, patients with diabetic nephropathy are known to have a higher risk of skeletal muscle atrophy [46]. Unfortunately, we did not estimate glomerular filtration rate. However, none of the study participants had clinical symptoms of kidney disease. Third, data on muscle strength were not available. It is possible that some participants had dynapenia (impaired strength without evident skeletal muscle mass loss). A higher risk of metabolic syndrome in individuals with dynapenia has been reported [47] and this has been described as an early stage of sarcopenia [48]. Fourth, our study did not investigate muscle fiber composition. An earlier study found a correlation between paraspinous muscle fat deposition and HOMA-IR (but not between psoas muscle fat deposition and HOMA-IR) in T2DM [15]. The paraspinous muscle group has a greater type II to type I fibers’ ratio compared with the psoas muscle. While type II fibers have 2–3 times less fat than type I fibers in physiological conditions, type II fibers have a lower insulin sensitivity than type I fibers. Although a review on the topic has suggested no changes in muscle fiber composition in T2DM individuals [49], it is worth investigating if this holds true in the PPDM setting. Last, the study design was cross-sectional; hence, the temporal relationship between skeletal muscle size and diabetes is still unclear. Some prospective studies have investigated changes in muscle mass in individuals with diabetes [50,51], with conflicting findings. A four-year follow-up study found an increase in lean mass in individuals with new-onset prediabetes and diabetes compared with normoglycemia individuals [50], whereas a six-year follow-up study observed a decrease in skeletal muscle mass in elderly T2DM individuals compared with normoglycemia individuals [51]. Given this discrepancy, the relationship between skeletal muscle mass and diabetes appears to be convoluted, and a bidirectional theory linking diabetes and reduced fat-infiltrated skeletal muscle mass in a self-perpetuating cycle has been proposed [52].

In conclusion, PMV is significantly lower in individuals with diabetes after pancreatitis compared with healthy controls. Furthermore, PMV is significantly directly associated with the Matsuda index of insulin secretion and is significantly inversely associated with SMFD. The relationship between skeletal muscle size and composition and PPDM warrants purposely designed prospective studies.

## Figures and Tables

**Table 1:** Characteristics of the study groups.

Characteristic	Diabetes After AP (*n* = 26)	Prediabetes After AP (*n* = 54)	Normoglycemia After AP (*n* = 33)	Healthy Controls (*n* = 39)	*p*
**Age (years)**	59 (49–69)	57 (46–68)	54 (46–62)	49 (39–59)	0.068
**Sex**					0.012
**Men**	21 (80.8%)	39 (53.7%)	17 (51.5%)	19 (48.7%)	
**Women**	5 (19.2%)	25 (46.3%)	16 (48.5%)	20 (51.3%)	
**V/S Fat Volume Ratio**	0.95 (0.70–1.20)	0.70 (0.35–1.05)	0.50 (0.35–0.65)	0.36 (0.21–51)	**<0.001**
**HbA1c (mmol/mol)**	52.0 (40.6–63.4)	39.0 (36.8–41.2)	34.0 (32.1–35.9)	33 (30.5–35.5)	**<0.001**
**Fasting Plasma Glucose (mmol/L)**	7.60 (6.65–8.55)	5.50 (5.10–5.90)	4.90 (4.65–5.15)	4.75 (4.30–5.20)	**<0.001**
**Physical Activity**					0.670
**Inactive**	4 (23.5%)	10 (22.7%)	10 (35.7%)	7 (26.9%)	
**Active**	13 (76.5%)	34 (87.3%)	18 (64.3%)	19 (73.1%)	
**Smoking status**					0.093
**Never**	12 (50.0%)	20 (37.8%)	13 (39.4%)	22 (57.9%)	
**Former**	9 (37.5%)	20 (37.8%)	13 (39.4%)	11 (28.9%)	
**Light**	2 (8.3%)	6 (11.3%)	3 (9.1%)	5 (13.2%)	
**Moderate/ heavy**	1 (4.2%)	7 (13.2%)	4 (12.1%)	0 (0%)	
**Alcohol consumption (U/week)**	21 (0–102)	24 (0–93)	96 (0–258)	0 (0–2)	0.152
**ACCI**	2.0 (0.5–3.5)	2.0 (0.5–3.5)	1.0 (0–1)	0.0 (0.0–0.5)	**0.001**
**Pancreatic amylase (U/L)**	25.0 (15.3–32.7)	14.1 (7.6–20.6)	14.1 (9.1–19.1)	20.6 (12.1–29.1)	0.073

Abbreviations: AP: Acute pancreatitis, HbA1c: Glycated hemoglobin, V/S: Visceral to subcutaneous fat volume ratio, ACCI: Age-adjusted Charlson index. Footnotes: Data are presented as median and interquartile range or counts frequency and percentages. *P* values were from analysis of variance (continuous variables) and chi-squared (categorical variables) tests. Statistically significant (*p* < 0.05) differences are shown in bold.

**Table 2:** Differences in psoas muscle volume between the post-pancreatitis groups and the healthy controls group.

Study Groups	Model 1			Model 2			Model 3			Model 4			Model 5		
	β	SE	*p*	β	SE	*p*	β	SE	*p*	β	SE	*p*	β	SE	*p*
**Normoglycemia**	−24.135	16.626	0.149	−24.264	11.095	**0.030**	−23.420	12.64	0.066	−23.254	12.940	0.075	−21.626	11.397	0.060
**Prediabetes**	3.097	14.739	0.834	−16.824	10.092	0.098	−20.266	12.125	0.098	−16.587	12.705	0.195	−12.179	10.799	0.261
**Diabetes**	−13.546	17.783	0.447	−42.619	12.368	**0.001**	−35.860	15.494	**0.023**	−36.667	16.089	**0.025**	−30.015	14.038	**0.034**

Abbreviations: V/S: Visceral/subcutaneous (fat volume ratio), ACCI: Age-adjusted Charlson comorbidity index. Footnotes: Data are presented as beta coefficients (i.e., the median difference in total psoas volume between each post-pancreatitis group and healthy controls group) and standard errors, from linear regression models. Statistically significant (*p* < 0.05) differences are shown in bold. Model 1: Unadjusted model; Model 2: Adjusted for age and sex; Model 3: Adjusted for age, sex, V/S fat volume ratio, and physical activity; Model 4: Adjusted for age, sex, V/S fat volume ratio, physical activity, smoking status, and alcohol consumption; Model 5: Adjusted for age, sex, V/S fat volume ratio, physical activity, smoking status, alcohol consumption, ACCI, pancreatic amylase, and use of insulin. Statistically significant (*p* < 0.05) differences are shown in bold.

**Table 3:** Associations between psoas muscle volume and indices of insulin secretion and sensitivity.

Insulin Traits	Model 1			Model 2			Model 3			Model 4			Model 5		
	β	SE	*p*	β	SE	*p*	β	SE	*p*	β	SE	*p*	β	SE	*p*
Indices of insulin secretion															
**HOMA-ß**	0.026	0.109	0.816	0.020	0.077	0.795	0.035	0.079	0.663	0.068	0.073	0.355	0.039	0.075	0.605
**Stumvoll index**	0.000	0.001	0.540	0.000	0.000	0.486	0.000	0.000	0.606	0.000	0.000	0.993	0.000	0.000	0.923
**Insulinogenic index 30′**	1.731	8.883	0.846	−1.613	5.893	0.785	−4.068	5.574	0.469	−3.810	5.562	0.497	−2.840	6.268	0.653
**Insulinogenic index 60′**	11.858	8.787	0.183	5.398	6.427	0.405	4.670	6.084	0.446	−2.496	5.883	0.674	−3.227	6.025	0.595
**Indices of insulin sensitivity**															
**HOMA-IS**	−9.835	16.762	0.559	−9.260	11.869	0.438	−8.596	11.957	0.475	−8.370	10.850	0.444	−5.824	13.911	0.677
**Raynaud index**	7.307	7.116	0.307	5.247	4.718	0.269	3.877	4.763	0.418	1.947	4.477	0.665	2.133	4.527	0.639
**1/fasting insulin**	42.089	40.987	0.307	30.225	27.174	0.269	22.332	27.437	0.418	11.216	25.786	0.665	12.283	26.075	0.639
**Matsuda index**	0.959	0.971	0.327	1.801	0.619	**0.005**	1.560	0.630	**0.016**	1.359	0.581	**0.022**	1.633	0.612	**0.010**

Abbreviations: V/S: Visceral/subcutaneous (fat volume ratio), ACCI: Age-adjusted Charlson comorbidity index. Footnotes: Data from the overall cohort are presented as beta coefficients (i.e., the median difference in PMV between each post-pancreatitis group and healthy controls group) and standard errors, from linear regression models. Statistically significant (*p* < 0.05) differences are shown in bold. Model 1: Unadjusted model; Model 2: Adjusted for age and sex; Model 3: Adjusted for age, sex, V/S fat volume ratio, and physical activity; Model 4: Adjusted for age, sex, V/S fat volume ratio, physical activity, smoking status, and alcohol consumption; Model 5: Adjusted for age, sex, V/S fat volume ratio, physical activity, smoking status, alcohol consumption, ACCI, pancreatic amylase, and use of insulin. Statistically significant (*p* < 0.05) associations are shown in bold.

**Table 4:** Associations between psoas muscle volume and fat depositions.

		Liver Fat	Intra-Pancreatic Fat	Skeletal Muscle Fat
Model 1	β	0.133	0.259	−4.794
	SE	0.609	2.891	0.749
	*p*	0.827	0.929	**<0.001**
Model 2	β	−0.518	−1.725	−2.321
	SE	0.418	2.093	0.764
	*p*	0.218	0.411	**0.003**
Model 3	β	0.005	2.430	−2.514
	SE	0.537	2.379	0.841
	*p*	0.397	0.304	**0.003**
Model 4	β	0.322	2.675	−2.590
	SE	0.563	2.422	0.833
	*p*	0.569	0.272	**0.002**
Model 5	β	−0.001	1.518	−3.080
	SE	0.480	2.257	0.761
	*p*	0.999	0.503	**<0.001**

Abbreviations: V/S: Visceral/subcutaneous (fat volume ratio), ACCI: Age-adjusted Charlson comorbidity index. Footnotes: Data from the overall cohort are presented as beta coefficients (i.e., the median difference in total psoas volume between each post-pancreatitis group and healthy controls group) and standard errors, from linear regression models. Statistically significant (*p* < 0.05) differences are shown in bold. Model 1: Unadjusted model; Model 2: Adjusted for age and sex; Model 3: Adjusted for age, sex, V/S fat volume ratio, and physical activity; Model 4: Adjusted for age, sex, V/S fat volume ratio, physical activity, smoking status, and alcohol consumption; Model 5: Adjusted for age, sex, V/S fat volume ratio, physical activity, smoking status, alcohol consumption, ACCI, pancreatic amylase, and use of insulin. Statistically significant (*p* < 0.05) associations are shown in bold.
